# Are Autistic Traits in the General Population Related to Global and Regional Brain Differences?

**DOI:** 10.1007/s10803-015-2441-6

**Published:** 2015-04-07

**Authors:** P. Cédric M. P. Koolschijn, Hilde M. Geurts, Andries R. van der Leij, H. Steven Scholte

**Affiliations:** Dutch Autism & ADHD Research Center, University of Amsterdam, Amsterdam, The Netherlands; Amsterdam Brain and Cognition, University of Amsterdam, Amsterdam, The Netherlands; University of Amsterdam, Weesperplein 4; R3.07, 1018 XA Amsterdam, The Netherlands; Dr Leo Kannerhuis, Amsterdam, The Netherlands

**Keywords:** Autistic traits, Gray matter volume, Cortical thickness, Surface area, Diffusion tensor imaging, Autism

## Abstract

There is accumulating evidence that autistic-related traits in the general population lie on a continuum, with autism spectrum disorders representing the extreme end of this distribution. Here, we tested the hypothesis of a possible relationship between autistic traits and brain morphometry in the general population. Participants completed the short autism-spectrum quotient-questionnaire (AQ); T1-anatomical and DWI-scans were acquired. Associations between autistic traits and gray matter, and white matter microstructural-integrity were performed on the exploration-group (N = 204; 105 males, M-age = 22.85), and validated in the validation-group (N = 304; 155 males, M-age = 22.82). No significant associations were found between AQ-scores and brain morphometry in the exploration-group, or after pooling the data. This questions the assumption that autistic traits and their morphological associations do lie on a continuum in the general population.

**Electronic supplementary material:**

The online version of this article (doi:10.1007/s10803-015-2441-6) contains supplementary material, which is available to authorized users.

## Introduction

It has been proposed that psychiatric symptoms, such as autism, lie on a continuum with normality (Constantino and Todd 2003). Several studies have reported that autistic traits are common in the general population (Ronald and Hoekstra 2011; Skuse et al. 2005). In addition, several authors have suggested that autism spectrum disorder (ASD) can be conceptualized as arising in individuals found at the extreme end of a normal distribution of autistic-like traits (Constantino and Todd 2003). One strategy to investigate this theoretical continuum is the use of endophenotypes. Although criteria for the validity of endophenotypic markers differ across studies, there is overall consensus that endophenotypes are quantitative, heritable, trait-related deficits typically assessed by laboratory-based methods rather than clinical observation (for overview see Bearden and Freimer 2006). The search for endophenotypes is based on the assumption that behavioral symptoms can be linked to neurobiological (and genetic) underpinnings both in the clinical population and the general population. Neuroimaging measurements have potential interest as endophenotypes for ASD, because these methods are typically repeatable, provide quantitative data, and may be more sensitive than behavioral observations to subtle brain changes. Indeed, several lines of evidence coming from twin and sibling studies, do suggest that structural brain abnormalities are present in unaffected co-twins and siblings though to a lesser degree than in people with ASD (Barnea-Goraly et al. 2010; Kates et al. 2004; Mitchell et al. 2009) which is in line with the endophenotypic view of ASD.

In the past few years an increasing number of neuroimaging studies have also investigated potential associations between autistic traits and (typically developing) controls in brain structure, using cross-sectional neuroimaging designs. Here we focus specifically on those studies using the autism-spectrum quotient AQ; (Baron-Cohen et al. 2001) as this instrument was designed specifically to measure variation in autistic traits in non-clinical samples, and has demonstrated good internal consistency and test–retest reliability in the Dutch population (Hoekstra et al. 2008). An overview of studies reporting associations between autistic traits, as measured with the AQ, and brain structure and function is presented in Table 1. Generally, these studies have reported relatively small associations with gray matter indices and AQ-scores in healthy subjects (larger and smaller volumes: Geurts et al. 2013; Saito et al. 2014) or no differences (Kosaka et al. 2010; Watanabe et al. 2014). With minor exceptions [smaller left inferior parietal lobule (Geurts et al. 2013); smaller insula (Saito et al. 2014)] these findings do not converge compared to meta-analytical findings of gray matter abnormalities in ASD (Cauda et al. 2011; Duerden et al. 2012; Nickl-Jockschat et al. 2012; Stanfield et al. 2008; Via et al. 2011). These discrepancies may be accounted for by participants’ age, because the studies presented in Table 1 all concern young adults while the meta-analyses in ASD report findings from childhood to adulthood. This argument is further supported by structural brain differences between children and adults with ASD, generally showing larger brain abnormalities in childhood compared to adulthood (Duerden et al. 2012). If brain abnordonemalities in ASD lessen with age (see also Raznahan et al. 2010), it might be that autistic traits in the healthy adult population may show only moderate associations with structural brain indices.

**Table 1:** Structural neuroimaging studies on autistic traits limited to autism-spectrum quotient in controls

	N	Sex %M	Age (SD)	Age range	AQ	AQ version	Measure	Results
*SMRI*								
Kosaka et al. (2010)	32 PDD-NOS 40	100	23.8 (4.2)	18–34	32.0 (5.7)	Full	VBM	Higher AQ score ⇒ *smaller* GM volumes of R insula and R IFG for whole group, but not in NC separately
	NC	100	22.5 (4.3)	17–32	17.1 (5.8)			
Von dem Hagen et al. (2011)	91 NC	41.8	25 (5)	18–42	16 (7)	Full	VBM/fMRI	Higher AQ scores ⇒ *smaller* WM volume in pSTS; AQ was correlated with extent of cortical deactivation near pSTS for contrast stroop > rest
Geurts et al. (2013)	85 NC	62.4	21.5 (2.4)	18–29	55.3 (17.2)	Full^a^	VBM	Higher AQ score ⇒ *larger* GM volume of L middle frontal gyrus; and *smaller* GM volume in L IFG; L central gyrus; PCC; and L inferior and superior parietal lobe.
Saito et al. (2014)	79 M NC		29.4 (4.2)	21–40	59.4 (11.4)	Full^a^	VBM	Lower AQ prosociality score ⇒ *smaller* R insula in *males*, and *lower* prosociality scores ⇒ *reduced* structural coupling of R insula with ventral ACC in *males*
	56 F NC		28.1 (4.4)	22–40	57.0 (13.6)			
Watanabe et al. (2014)	51 ASD	100	30.9 (8.2)	19–51	35.5 (5.3)	Full	SulcoGyral pattern	No association between sulcal subtype and AQ score
	55 NC	100	32 (7.1)	19–49	14.3 (5.8)			
*DTI*								
Iidaka et al. (2012)	30	46.7	22.5 (3.0)	n.a.	21.2 (6.2)	Full	DTI/fMRI	Higher AQ score ⇒ *larger* volume of connectivity between the STS and AMG, and with imagination sub-scale

*AQ* autism spectrum quotient, *PDD*-*NOS* pervasive developmental disorder not otherwise specified, *VBM* voxel-based morphometry, *NC* neurotypical controls, *M* males, *F* females, *L* left, *R* right, *ACC* anterior cingulate cortex, *AMG* amygdala, *GM* gray matter, *IFG* inferior frontal gyrus, *PCC* posterior cingulate cortex, (*p*)*STS* (posterior) superior temporal sulcus, *WM* white matter

^a^4-point scale of AQ

So far only one study reported autistic traits in a non-clinical sample and the association with white matter as measured with diffusion tensor imaging (DTI; Iidaka et al. 2012). Here the authors examined an a priori defined white matter fiber bundle associated with face-processing and reported increased white matter connectivity volume (between superior temporal sulcus and amygdala) with higher AQ-scores. Although it is difficult to embed this result in the current literature due to the specific fiber selection, the results do not overlap with volumetric white matter findings in ASD (Radua et al. 2011). Furthermore, it is important to note that the relationship between ASD symptomatology and white matter integrity in ASD is rather mixed, with equaling numbers of studies reporting associations or a lack thereof (Ameis and Catani 2014). These variable results have been related to the heterogeneity of the disorder, small sample sizes, and different methodologies (Ameis and Catani 2014).

The purpose of the current study was to examine the association between autistic traits in young non-autistic adults with a variety of structural brain indices: gray matter volume, cortical thickness, surface area, structural coupling and DTI parameters. To this end, we used an exploration-validation design in two large independent samples (Exploration N = 204; Validation N = 304), stratified for age, sex, and level of education. The exploration strategy allowed us to explore brain-behavior relationships without the need to correct for multiple comparisons. The Validation study evaluated these brain-behavior relationships with appropriate statistical measures to account for multiple comparisons. A (significant) confirmation of these brain-behavior relationships in the independent sample indicates replication of these findings. If brain-behavior associations were not confirmed in the Validation study, this suggests that there’s no association between autistic traits and our structural brain indices.

The first goal of this study was, therefore, to elucidate the relationship between autistic traits and a number of gray matter indices. First, we aimed to replicate our VBM findings from an earlier independent study (Geurts et al. 2013) (Table 1). Second, we aimed to extend these findings to associations in cortical thickness, surface area and structural coupling. Cortical thickness findings in ASD generally show atypical brain maturation (Raznahan et al. 2010), thinner cortical regions (Scheel et al. 2011; Wallace et al. 2010), and increased frontal lobe thickness has also been reported in adults with ASD (Ecker et al. 2013). In addition, positive associations between scores on the Autism Diagnostic Interview (ADI-R; Lord et al. 1994) and frontal and parietal thickness have been reported (Ecker et al. 2013). Based on these findings in ASD, we expected associations between autistic traits and cortical thickness in neurotypicals regardless of the direction. Reports on surface area (SA) in young adults measures have revealed reduced SA, primarily in orbito-frontal cortex and posterior cingulum (Ecker et al. 2013), or no differences between ASD and controls (Haar et al. 2014; Raznahan et al. 2010; Richter et al. 2015; Wallace et al. 2013). Here we didn’t expect SA differences to be related to autistic traits.

Finally, structural coupling was assessed to look at gray matter networks. A large number of studies have postulated that autism is associated with abnormal brain wiring (Kana et al. 2011; Vissers et al. 2012). Although most studies have used diffusion tensor imaging (DTI) or resting-state fMRI to assess structural and functional connectivity (Vissers et al. 2012), both structural covariance and structural coupling measures have also been used investigate brain networks. The biological nature of gray matter morphological networks remains unclear, but it has been suggested that covarying brain regions indicate synchronized maturation (Alexander-Bloch et al. 2013) or common experience-related plasticity (Mechelli et al. 2005). In ASD, the salience network appears to be undersized, whereas the default mode network (DMN) demonstrates both under- as well as over-connected components, some outstretching typical DMN network topology (Zielinski et al. 2012). Graph-theoretical methods have also been applied to gray matter networks in ASD. Reduced modularity (highly connected nodes within modules compared to between modules) has been reported in autistic children compared to controls. Furthermore, enlarged frontal correlation strength (within module) has been found, while long distance connections between frontal and other lobes demonstrated reduced correlation strength (Shi et al. 2013). Here we explored structural covariance based on cortical thickness and gray matter volume in neurotypical adults and the association with autistic traits.

The second goal of this study was to examine the relationship between white matter, i.e. fractional anisotropy (FA; white matter integrity and the directional dependency of water diffusion in the brain), and autistic traits. Prior studies in ASD generally indicate reduced FA-values most consistently found in regions such as the corpus callosum, cingulum, and parts of the temporal lobe (Travers et al. 2012). Although autistic symptom severity has been associated with FA-values, there’s no consensus on directionality and further interpretation is also hampered by (relatively) small samples (Travers et al. 2012). This led us to hypothesize that only weak or no associations were to be expected between autistic traits and FA-values.

## Methods

### Participants

In this study we used two datasets. The Exploration sample was used to explore associations between AQ-scores and structural brain indices. The Validation sample was used to test the generalizability of the findings based on the first set.

A total of 508 participants between ages 20–26 years were recruited from different backgrounds and were representative of the Dutch population in several ways (IQ, sex, socioeconomic background) and took part in a larger population based study (see Pinto et al. 2013). Individuals were randomly assigned to the two independent samples, with the restriction that both samples were stratified for age, sex and level of education (low, medium and high as determined on the basis of the highest level of education the subject participated in; Table 2). Participants were only excluded from the study on the basis of a neurological disorder, schizophrenia and epilepsy. Some of the participants had a self-reported ASD-diagnosis (Exploration: N = 10, 4.9 %; Validation: N = 6, 2 %). In addition, some of the participants also had clinical scores on the AQ-28 (Exploration: N = 12, 6 %; Validation: N = 21, 7 %; clinical cut-off: AQ > 70; Hoekstra et al. 2011) that might be indicative for ASD-related problems. There was limited overlap between self-reported diagnosis and clinical cut-off scores based on the AQ-28 (concordance Exploration sample: 1 out of 12; Validation sample 3 out of 21). Excluding those subjects with a self-reported ASD-diagnosis didn’t change the results. Therefore, all participants were included in subsequent analyses.

**Table 2:** Demographics of the exploration and validation samples

	Exploration sample	Validation sample	Statistics
	N = 204	N = 304	F/χ^2^ (*p* value), effect size
Age	22.85 (1.7)	22.82 (1.73)	F = 0.02 (0.88); η^2^ = 4.9E10^−5^
Sex	105 M (51 %)	155 M (51 %)	*χ* ^*2*^ = 0.01 (0.92); Φ = 0.005 (0.92)
Education (N)			*χ* ^*2*^ = 0.07 (0.97); Φ = 0.01 (0.97)
Low	25 (12 %)	35 (12 %)	
Middle	87 (43 %)	131 (43 %)	
High	92 (45 %)	138 (45 %)	
Handedness (N)			*χ* ^*2*^ = 0.71 (0.40); Φ = 0.04 (0.40)
Left	22 (11 %)	26 (9 %)	
Right	182 (89 %)	278 (91 %)	
AQ^a^ (Mean, SD, range)			
Total	55.63 (8.96) [33–80]	57.05 (8.70) [32–91]	F = 3.18 (0.08); η^2^ = 0.006
Broad factor social behavior	45.69 (7.80) [27–70]	47.31 (7.60) [25–75]	F = 5.43 (0.02); η^2^ = .01^b^
Social skills	12.39 (3.65) [7–28]	13.02 (3.67) [7–24]	F = 3.55 (0.06); η^2^ = 0.007
Routine	8.39 (1.84) [4–13]	8.63 (1.73) [4–14]	F = 2.16 (0.14); η^2^ = 0.004
Switching	8.87 (1.85) [4–13]	9.08 (2.00) [4–15]	F = 1.44 (0.23); η^2^ = 0.003
Imagination	16.03 (3.38) [8–23]	16.58 (3.39) [8–31]	F = 3.19 (0.08); η^2^ = 0.006
Numbers/patterns	9.94 (3.10) [5–19]	9.74 (3.21) [5–20]	F = 0.48 (0.49); η^2^ = 0.01

*M* males

^a^4-point scale

^b^A common side effect of working with large samples is the tendency that small between group differences become statistically significant, but may be unimportant. Here we report effect sizes to illustrate the magnitude of these effects. The difference in mean score on the broad factor social behavior subscale is significantly different between groups; however, the effect size shows that the magnitude is rather small (a value of η = 0.02 is considered small, here we report an even lower value: of η = 0.01). It should be noted that in all of our analyses we used the aggregated score of the AQ, i.e. we didn’t examine associations based on AQ subscales in any of our analyses

Participants gave written informed consent for the study and received fixed payment for participation. The internal review board from the University of Amsterdam approved the study.

**Fig. 1:**
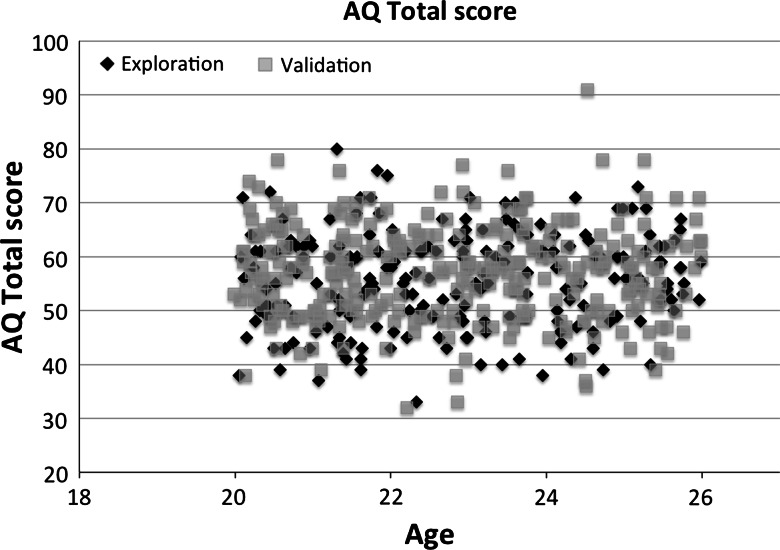
Scatterplot of the total scores on the AQ-28 for the Exploration sample (*black diamonds*) and Validation sample (*gray squares*)

To measure autistic traits, participants completed the Dutch short Autism-Spectrum Quotient (AQ-28; Hoekstra et al. 2011), which is a 28-item self-report measure of ASD-related traits based on Dutch and British control samples and a sample with people with an Asperger syndrome diagnosis. The AQ-Short consists of two higher-order factors assessing ‘social behavioral difficulties’ (communication items show very high correlations, and were therefore removed) and ‘a fascination for numbers/patterns’. The correlation with the full-scale AQ (50-item list) is very high (*r* between 0.93 and 0.95; Hoekstra et al. 2011). All items were answered on a four-point scale (0–3). A higher score on the AQ-28 means that more severe ASD symptoms are present (see Table 2, potential score range is 0–150). Average scores from the validation study of three independent control samples (Dutch and British) were similar compared to our samples (Mean AQ-score: 52–60 in (Hoekstra et al. 2011); Mean AQ-score Exploration = 55.6; Mean AQ-score Validation = 57.1). Test–retest and inter-rater reliability of the AQ-28 are good (Hoekstra et al. 2011). Figure 1 displays the distribution and range of AQ-scores for the Exploration and Validation sample (W’s = 0.99; *p*’s > 0.2).

### Data Acquisition

All participants were scanned in a single-session on a 3-Tesla whole body Philips Achieva TX MRI system (Best, The Netherlands). Three high-resolution T1-weighted were obtained: 3D-T1-weighted scan (3D-TFE, T1-weighted images TE 3.8 ms; TR 8.2 ms; Flip Angle (FA) 8°; 160 sagittal slices of 1 mm; field of view (FOV), 256^2^ mm; reconstruction matrix 256^2^), total duration 18 min, and were averaged to increase signal-to-noise-ratio. Since diffusion data have relatively low signal-to-noise ratio, we also collected three recordings in which we obtained diffusion weighted imaging (DWI) measurements (DWI-SE; TE 73.36 ms; TR 6312 ms; FA 90°; 60 transversal slices of 2 mm; FOV 224^2^mm; reconstruction matrix 112^2^, 32 directions, b0 = 1000 s/mm^2^), total duration 14.35 min. Additional scans were acquired, but are not reported here. Subjects were in the scanner for 43 min of actual recording time while a typical scanning session lasted 60 min.

### Image Processing and Analysis

#### Voxel-Based Morphometry

The VBM-analysis was performed with FSL software v.5.0 (www.fmrib.ox.ac.uk/fsl/). T1 scans were averaged after brain extraction but before brain segmentation (no special normalization). First, structural images were brain-extracted and gray matter-segmented before being registered to the MNI152 standard space using non-linear registration (Andersson et al. 2007). The resulting images were averaged and flipped along the x-axis to create a left–right symmetric, study-specific gray matter template. Second, all native gray matter images were non-linearly registered to this study-specific template and “modulated” to correct for local expansion (or contraction) due to the non-linear component of the spatial transformation. The modulated gray matter images were then smoothed with an isotropic Gaussian kernel with a sigma of 4 mm. Finally, voxel-wise GLM was applied using permutation-based non-parametric testing.

#### Cortical Thickness and Cortical Gray Matter Volume

Cortical reconstruction and volumetric segmentation was measured automatically using FreeSurfer 5.3 (http://surfer.nmr.mgh.harvard.edu/, Dale et al. 1999; Fischl and Dale 2000). Details of the surface-based cortical reconstruction procedures have been extensively documented previously (Dale et al. 1999; Fischl and Dale 2000; Fischl et al. 2004; Segonne et al. 2004). Briefly, the FreeSurfer pipeline performs motion correction on the T1-images, automatically removes non-brain tissues (Segonne et al. 2004), transforms volumetric data to a common atlas, performs intensity normalization and topology correction (Fischl et al. 2004; Segonne et al. 2007) and defines the boundaries of the gray/white matter and pial surface (Dale et al. 1999; Fischl and Dale 2000). For the purposes of the current study, automated image surfaces and segmentations were inspected and screened for quality control but were not manually edited, in order to maintain the objectivity of results. Intracranial volume was determined by a validated automated method known to be equivalent to manual intracranial volume estimation (Buckner et al. 2004). Three subjects (Validation sample) were excluded due to segmentation failures in FreeSurfer resulting in 301 subjects suitable for analyses.

#### DTI

All DTI preprocessing and analyses were conducted using FSL tools (www.fmrib.ox.ac.uk/fsl). Voxel-wise statistical analysis of the FA-data was carried out using TBSS (Tract-Based Spatial Statistics, Smith et al. 2006). First, FA images were created by fitting a tensor model to the raw diffusion data using FMRIB’s Diffusion Toolbox (Behrens et al. 2003) and then brain-extracted using BET (Smith 2002). DWI sequences were not averaged but treated as a recording of three times the length of any single DWI recording. Eddy current/motion correction therefore included accounting for any differences between the (successive) DWI recordings (Jenkinson and Smith 2001). All non-brain data were discarded, and images were aligned to MNI152 standard space and were visually inspected to confirm a close registration. Next, the mean FA-image was created and thinned to create a mean FA-skeleton, which represents the centers of all tracts common to the group. Each subject’s aligned FA-data was then projected onto this skeleton and the resulting data fed into voxel-wise cross-subject statistics.

### Statistical Analyses

#### VBM and DTI

In the VBM-analysis we wanted to isolate voxels that were correlated with the AQ-28 total score in the Exploration sample and evaluate the overlap of these correlations in the Validation sample. We tested these two matters in two separate steps. First, AQ-28 total scores correlation inferences were examined using permutation-based non-parametric testing on the Exploration sample, with age, sex, handedness, level of education and intracranial volume as nuisance factors. Next, regions of interest were extracted from the resulting statistical maps using a minimum cluster size of 100 voxels (for a similar procedure see Rouw and Scholte 2010) that surpassed a threshold of *p* < 0.05 (uncorrected for multiple comparisons). Second, we tested if these clusters were correlated with AQ-28 total score in the Validation sample using corrections for multiple comparisons (Bonferroni, *p* < 0.05/number of ROIs).

The same approach was used for the FA-analyses, albeit with a lower minimal cluster size threshold: 50 voxels.

#### Cortical Thickness and Cortical Gray Matter Volume

The cortical thickness, cortical volume and surface area data were averaged across participants in the spherical coordinate system after smoothing (FWHM 15 mm), so that surface areas with significant differences of mean cortical thickness differences and the AQ-scores could be overlaid in statistical difference maps (using *t*-statistics) for the Exploration study. Vertex-wise analyses were performed using a general linear model approach. We addressed differences in cortical thickness, gray matter volume, and pial surface area (gray matter surface area) for the Exploration sample and AQ-score, with age, sex, handedness, level of education, and intracranial volume as nuisance factors. Due to the large number of tests, differences were reported as significant below a FDR-corrected *p* value of 0.05. In case of significant findings in the Exploration study, the ROIs were extracted and tested for in the Validation study using stringent Bonferroni correction for multiple comparisons.

#### Structural Gray Matter Coupling

Freesurfer was used to parcellate the cortical gray matter into 68 regional labels (suppl. Table 1) and we extracted both cortical thickness as well as cortical gray matter volume. For the volumetric analyses, the cerebellum and subcortical regions of interest (ROIs; amygdala, caudate nucleus, hippocampus, globus pallidum, nucleus accumbens, putamen and thalamus) were also included. To map structural covariance networks between each pair of ROIs, partial correlation coefficients were calculated for every pair of regions across subjects (for cortical thickness and gray matter volume), creating a 68 × 68, group correlation matrix (where N is the number of ROIs) for the cortical thickness and an 84 × 84, group correlation matrix for the volumetric analyses. To examine the influence of autistic traits on these correlations, two correlation matrices were built for each measure for both the Exploration and Validation sample: (1) controlling for age, sex, handedness, intracranial volume and level of education; (2) adding AQ-28-score as additional control variable.

To the test whether AQ-28-score influences structural coupling, Fisher’s r-to-z transformations were applied to obtain a comparable Z-value instead of the original r, and then a difference of any paired Z-value (between matrices with and without controlling for AQ-28-score) calculated with |ΔZ| > 1.96 (*p* < 0.05, standard normal distribution, 95 % confidence interval) was considered significant. In case of a significant influence of AQ-28-score on structural coupling, the same correlation-pairs were checked in the Validation sample, using the abovementioned approach.

For the correlation analysis, performed for all 68 × 68/2 = 2312 for thickness and 84*84/2 = 3528 for gray matter volume pairs of regions, we applied a Bonferroni correction to correct for multiple comparisons with *p* < 4 × 10^−4^ (0.05/2312) and *p* < 1.5 × 10^−5^ for cortical thickness and volume respectively.

## Results

All analyses have been run with and without those individuals with a self-reported diagnosis of ASD and/or AQ-scores above clinical cut-off (>70). Results remained similar for all analyses; therefore the results we report in this section include all individuals from both independent samples.

### VBM

Both positive and negative relationships between AQ-28-score and gray matter were found in the Exploration study. Specifically, higher AQ-28-scores were related to larger volumes of six brain regions in the Exploration study (see Table 3A first columns; note that here we don’t correct for multiple comparisons). To validate our findings, we extracted the mean gray matter volume of these six ROIs from the Validation sample, and performed partial correlations between these ROIs (controlling for age, sex, handedness, level of education and intracranial volume) and AQ-28-score controlling for multiple comparisons (*p* < 0.005). All of these correlations were small (<|0.07|) and none were significant (all *p*’s > 0.2; see Table 3A last columns), indicating that an association between autistic traits and gray matter volume is unlikely.

**Table 3:** Overview of brain regions resulting from correlation analyses of AQ total scores and gray matter volume estimates in validation sample (N = 304)

Brain area	Hemisphere	MNI Coordinates			kE	Validation sample	
		X	Y	Z		*r*	*p* value
(A) *Positive association AQ in Exploration sample*							
Precuneus/lingual gyrus/superior parietal lobule^a^	R/L	24	−58	−2	15,240	0.022	0.705
Frontal pole	R	44	50	8	7955	0.003	0.965
Precentral gyrus	L	−56	0	16	4654	0.074	0.203
Frontal pole	L	−48	44	8	2569	−0.066	0.259
Supramarginal gyrus	R	62	−28	52	214	0.026	0.655
Precentral gyrus	R	32	−16	72	181	0.011	0.851
(B) *Negative association AQ in Exploration sample*							
Hippocampus/parahippocampal gyrus^a^	L/R	−14	−18	−28	5864	−0.088	0.128
Postcentral gyrus	R	6	−34	62	347	0.003	0.962
Precuneus cortex	R	26	−62	18	220	0.063	0.281
Insular cortex	R	32	−10	18	180	0.006	0.922

*L* left, *R* right, *kE* cluster size mm^3^, *r* correlation with validation sample, *p value p* value of correlation with validation sample

^a^In these cases the ROI spanned several anatomical regions

Four brain regions showed negative associations with AQ-28-score in the Exploration study, indicating smaller brain volumes with higher AQ-28-scores (see Table 3B). Following the same procedure as for the positive associations; gray matter volume estimates were extracted from the Validation sample and partial correlations were performed with AQ-28-score using the same control variables. Again, only small (<|0.09|) non-significant associations were found in the Validation sample (Table 3B last columns), which suggests no association between AQ-28-score and gray matter volume.

### DTI

There were no relationships between AQ-28-score and FA-values in the Exploration study. Therefore we pooled the data (N = 508) and reran the analyses with threshold free cluster enhancement and corrected for multiple comparisons with family wise error correction (*p* < 0.05). No significant associations were found.

### Whole-Brain Vertex-Wise Analyses

Vertex-wise GLM was performed to test the association between AQ-28-scores and cortical thickness, gray matter volume, and pial surface area. No significant associations were found between autistic traits and any of the gray matter indices in the Exploration sample. Similar to the DTI approach, we combined our samples resulting in N = 505 (three subjects were excluded in FreeSurfer analyses), and test the relationship between AQ-28-score and gray matter indices after controlling for multiple comparisons (FDR: *p* < 0.05). Again, no significant relationships were found with cortical thickness, gray matter volume and pial surface area.

### Structural Coupling

#### Cortical Thickness Coupling

**Fig. 2:**
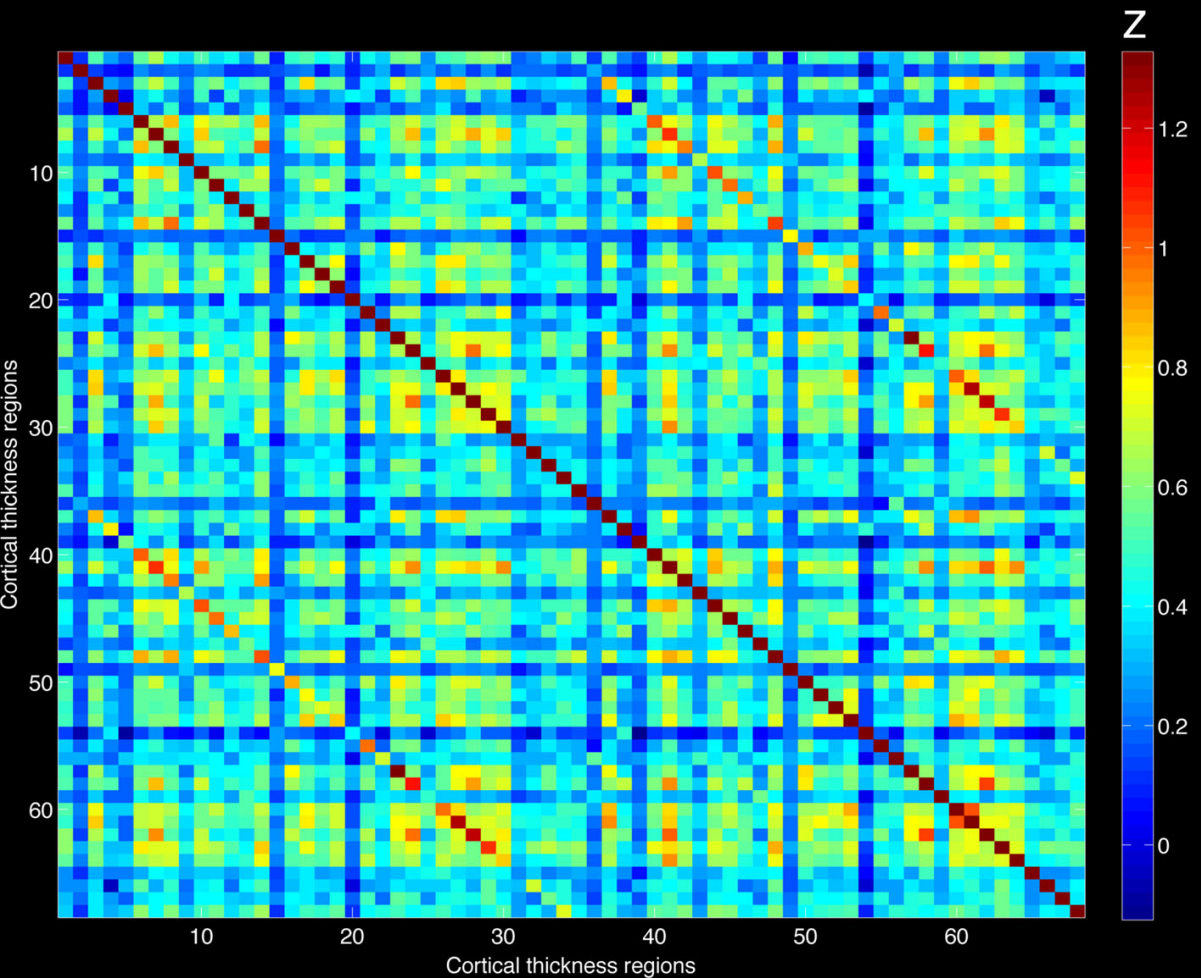
Cortical thickness coupling. The part above the diagonal represents the *Z*-values of the partial correlations controlled for AQ-28 (and age, sex, handedness, level of education and intracranial volume), the lower part of the diagonal represents the *Z*-values of the partial correlations controlled for age, sex, handedness, level of education and intracranial volume (see suppl. Table 1 for denotation of numbers)

In the Exploration study, controlling for AQ-28-score in the partial correlations, did not alter cortical thickness correlations (all |ΔZ’s| < 0.5). This indicates that symptom severity does not influence structural coupling in cortical thickness. The almost identical values are shown in Fig. 2: the upper part represents the Z-values of the partial-correlations controlling for AQ-28 (and age, sex, handedness, level of education and intracranial volume), the lower part represents the Z-values of the partial-correlations controlled for age, sex, handedness, level of education, and intracranial volume (see suppl. Table 1 for denotation of numbers).

The lack of AQ-28-score associations on structural coupling in the Exploration group, made us decide to pool the data (N = 505). However, no significant associations were found (all |ΔZ’s| < 0.5).

#### Gray Matter Volume Coupling

Similar to the cortical thickness coupling analyses, AQ-28-score did not influence gray matter volume coupling in the Exploration study (all |ΔZ’s| < 0.5) or the full sample [N = 505; (all |ΔZ’s| < 0.5].

## Discussion

In this large exploration-validation study, we investigated if autistic traits in neurotypical adults were associated with a comprehensive series of structural brain indices. In contrast to our hypotheses, no evidence was found for any relationships between individual differences in behavior and brain anatomy. This was further demonstrated by lack of brain-behavior associations in the combined sample of N = 508. Accordingly, these results do not provide evidence for the presumed continuum of autistic traits and associated morphological differences in the general population.

Although most initial reports found significant associations between autistic traits and regional brain volumes (see Table 1), we failed to show such an association in the largest study to date on autistic traits and brain structure. Our results are in part consistent with one study demonstrating AQ-brain volume associations in a sample consisting of people with PDD-NOS, but not in the control group (Kosaka et al. 2010). Similarly, Von dem Hagen and colleagues didn’t report associations between gray matter volume and autistic traits after correction for multiple comparisons (von dem Hagen et al. 2011). There are also a number of differences between our assessment and those reported in Table 1. First, we used an exhaustive assessment of structural brain indices comprising VBM, and also cortical thickness, cortical volume, surface area, gray matter coupling and FA-values. Direct comparisons between studies are limited to VBM results only, because the other measures were not taken into account and thus warrant replication. Second, we used the short version of the AQ (28 items) whereas the other studies used the full version (50 items). However, the short version has proven to demonstrate high sensitivity and specificity in the Dutch and British population, with high correlations with the full-scale version (*r*’*s* between 0.93 and 0.95) and the age-range on which the questionnaire was validated matches those from our sample (Hoekstra et al. 2011).

There are several possible interpretations for the absence of our findings. Our participants had relatively low scores on the AQ-28, despite similar variance in AQ-scores compared to earlier studies reporting brain-behavior associations (full AQ; Geurts et al. 2013; Saito et al. 2014), and to the validation study of the abbreviated AQ (Hoekstra et al. 2011).

An alternative interpretation is that gray and white matter abnormalities are only present in ASD and relatives, but are not associated with autistic traits in the general population (Kates et al. 2004, 2009; Segovia et al. 2014). This would suggest that clinical phenomena associated with ASD do not lie on a continuum with normality. Such a conclusion may be an oversimplification and warrants a detailed assessment. A (statistical) relationship between specific brain measures and autistic traits depends upon (1) sample size; and (2) the measure being studied. In case of the first, we believe that our approach, using an exploration-validation design with two large independent samples, provided sufficient power to detect possible brain-behavior associations if these were present. In case of the latter, there are a number of issues that need to be discussed.

We should separate the term “measure” as mentioned above into our neuroimaging measures and the autistic traits measure. In various neuroscience research fields, including psychiatric disorders, neuroimaging is considered to be a useful tool for the discovery of neuroimaging endophenotypes (e.g. Prasad and Keshavan 2008; Rijsdijk et al. 2010). Furthermore, this technique has proven the ability to allow identification of abnormal brain morphometry or activity in vivo that are predictive or associated with the development of a disorder/condition (Bearden and Freimer 2006; Glahn et al. 2007). By combining different modalities of structural neuroimaging, we believe that our approach employing standard, validated and accepted methodology (FSLVBM, FreeSurfer and TBSS) yielded reliable results.

As discussed previously, the AQ was developed to examine autistic traits in non-autistic individuals (Baron-Cohen et al. 2001). It is possible that the correspondence of autistic symptoms measured with the AQ(-28) in ASD and neurotypicals is not one-to-one. This was recently demonstrated in a study examining the AQ-28 in both individuals with ASD and controls. The authors showed that variables of the short AQ measure the same latent traits across ASD and control groups, but lack of scalar invariance (Murray et al. 2014). This means that equal observed scores on the AQ-28 do not necessarily imply equal levels of autistic traits (or severity) in an individual drawn from an ASD versus a non-autistic population. This has implications in the generalizability of AQ-related neuroimaging findings in the general population to those in ASD, and hence, lacks in the current practice the potential as an endophenotype.

Continuing along this line, the relationship between AQ and diagnosis instruments in ASD, such as the ADI-R (Lord et al. 1994) and Autism Diagnostic Observation Schedule (ADOS; Lord et al. 1989) has shown to be relatively low (Brugha et al. 2012; Ketelaars et al. 2008) compared to, for example, the SRS (social responsiveness scale) (Bolte et al. 2011). Moreover, it has been suggested that despite the fact that the AQ is a reasonably valid self-report measure, the SRS (Constantino et al. 2003) and the Broad Autism Phenotype Questionnaire (BAPQ; Hurley et al. 2007) may be more useful to assess autistic traits in the general population (Ingersoll et al. 2011). It should be noted that in a recent comprehensive comparative study on autistic trait questionnaires both the AQ and SRS (for adults) showed poor internal consistency and discriminant validity (Nishiyama et al. 2014). Thus, use of the AQ-28 for autistic traits and structural neuroimaging endophenotypes may not be beneficial in search of a valid imaging endophenotype.

To date, no studies have examined autistic traits measured by different questionnaires or self-reports and combined these with neuroimaging measures. So far, only two reports from one group have used the SRS in relation with brain morphometric measures. Wallace and colleagues described in a longitudinal study of typically developing children and young adults [age-range: 3.3–29.5], cortical thinning with greater autistic traits primarily in the bilateral superior and middle temporal regions (Wallace et al. 2012). In part of the same sample, higher SRS scores in areas associated with variation in the MET-gene, were related to reductions in cortical thickness in the same temporal regions, and pre- and post-central gyri, and bilateral anterior cingulate cortex, and right fronto-polar cortex (Hedrick et al. 2012). These findings showed considerable overlap with cortical thinning reported in ASD, but show almost no overlap with AQ-related brain associations as presented in Table 1. It should be noted that direct comparisons and interpretations between these studies is hampered by design (cross-sectional vs. longitudinal) and methodology (brain volumes vs. cortical thickness).

Finally, a number of various functional neuroimaging studies (fMRI, MEG, and NIRS) have associated autistic traits to task-related responses. Here too, findings are not in full accord with those in the ASD population. For instance, three studies reported positive associations between increased brain activity in posterior superior temporal sulcus and autistic traits [Stroop: (von dem Hagen et al. 2011); eye-gaze studies: fMRI (Nummenmaa et al. 2012), MEG (Hasegawa et al. 2013)], while in ASD inverse relationships for eye-gaze are reported (e.g. Pelphrey et al. 2005). Hence, also with functional neuroimaging no clear evidence is found for AQ-related associations with brain activity. For future studies it would be interesting to combine functional and structural neuroimaging (similar to von dem Hagen et al. 2011), as AQ-related brain associations may be easier to detect in task-relevant brain regions, as these may approximate behavior more closely, than whole brain structural neuroimaging studies. The most obvious approach would then be to examine the association between autistic traits and functional brain activity (during a task), and investigate whether brain structure (measured in terms of volume or cortical thickness) is associated with (1) functional brain activity; (2) autistic traits; and (3) mediates the relationship between those two.

The current study has a number of important strengths. First, the exploration-validation approach allowed us to examine brain-behavior relationships in two independent samples, both larger than those reported in the current literature. Second, we were able to integrate a large number of structural brain indices to ensure a comprehensive understanding of the associations between autistic traits and brain morphometry in the general population. Despite these strengths, our findings should be considered in light of some methodological considerations.

Our interpretations of the current study are limited to our narrow age-range. Similar to prior studies examining the association between AQ and brain structure, the AQ has been used solely in adults, no information is available for children and adolescents or elderly. Furthermore, we acknowledge the well-known issues of reliance on a self-report measure (see also Nishiyama et al. 2014).

Some might argue that individuals with a self-reported ASD-diagnosis (N = 10, N = 6 in Exploration and Validation sample respectively), and individuals with AQ-scores above the clinical cut-off (N = 12, N = 21 in Exploration and Validation sample respectively) should be excluded from analyses because this may bias our interpretation. However, excluding those individuals didn’t change our results, and given that including these individuals should, at least theoretically, increase the chance of finding an association between autistic traits and brain structure (which we do not report), we feel confident that our current findings are not due to inclusion of (possible) ASD-related diagnoses.

We recommend future studies to include a wider age-range to examine trait-related brain associations with age. In addition, we believe that autistic traits should be assessed by multiple measures, to gain a deeper understanding of the meaning of trait-related associations reported in ASD and non-autistic populations. Furthermore, we believe that, similar to standards in genetic research (e.g. Hirschhorn et al. 2002), declaring a brain structure-behavior association requires an exploration-validation (i.e. replication) design.

## Conclusions

Our results offer an unexpected contribution to the understanding of the neuroanatomical basis of autistic traits in the general population. Here we showed that in two large independent samples autistic traits were not associated with gray matter volume, cortical thickness, surface area, and structural coupling or white matter microstructural properties. This questions the assumption that autistic traits and their morphological associations do lie on a continuum in the general population.

## Electronic supplementary material

Supplementary material 1 (DOC 88 kb)
