# Supplementary material for: Are Autistic Traits in the General Population Related to Global and Regional Brain Differences?
Source: J Autism Dev Disord. 2015 Apr 7;45(9):2779–91. doi: 10.1007/s10803-015-2441-6 (PMC4553146; doi:10.1007/s10803-015-2441-6)
Supplement: Supplementary file 1 — Supplementary material 1 (DOC 88 kb) [file 10803_2015_2441_MOESM1_ESM.doc]

Supplemental Table 1. Brain regions for structural coupling

| **Number in matrix** | **Gray matter volumes** | **Cortical thickness** |
| --- | --- | --- |
| 1 | L Cerebellum | L bankssts |
| 2 | L Thalamus | L caudalanteriorcingulate |
| 3 | L Caudate | L caudalmiddlefrontal |
| 4 | L Putamen | L cuneus |
| 5 | L Pallidum | L entorhinal |
| 6 | L Hippocampus | L fusiform |
| 7 | L Amygdala | L inferiorparietal |
| 8 | L Accumbensarea | L inferiortemporal |
| 9 | R Cerebellum | L isthmuscingulate |
| 10 | R Thalamus | L lateraloccipital |
| 11 | R Caudate | L lateralorbitofrontal |
| 12 | R Putamen | L lingual |
| 13 | R Pallidum | L medialorbitofrontal |
| 14 | R Hippocampus | L middletemporal |
| 15 | R Amygdala | L parahippocampal |
| 16 | R Accumbensarea | L paracentral |
| 17 | L bankssts | L parsopercularis |
| 18 | L caudalanteriorcingulate | L parsorbitalis |
| 19 | L caudalmiddlefrontal | L parstriangularis |
| 20 | L cuneus | L pericalcarine |
| 21 | L entorhinal | L postcentral |
| 22 | L fusiform | L posteriorcingulate |
| 23 | L inferiorparietal | L precentral |
| 24 | L inferiortemporal | L precuneus |
| 25 | L isthmuscingulate | L rostralanteriorcingulate |
| 26 | L lateraloccipital | L rostralmiddlefrontal |
| 27 | L lateralorbitofrontal | L superiorfrontal |
| 28 | L lingual | L superiorparietal |
| 29 | L medialorbitofrontal | L superiortemporal |
| 30 | L middletemporal | L supramarginal |
| 31 | L parahippocampal | L frontalpole |
| 32 | L paracentral | L temporalpole |
| 33 | L parsopercularis | L transversetemporal |
| 34 | L parsorbitalis | L insula |
| 35 | L parstriangularis | R bankssts |
| 36 | L pericalcarine | R caudalanteriorcingulate |
| 37 | L postcentral | R caudalmiddlefrontal |
| 38 | L posteriorcingulate | R cuneus |
| 39 | L precentral | R entorhinal |
| 40 | L precuneus | R fusiform |
| 41 | L rostralanteriorcingulate | R inferiorparietal |
| 42 | L rostralmiddlefrontal | R inferiortemporal |
| 43 | L superiorfrontal | R isthmuscingulate |
| 44 | L superiorparietal | R lateraloccipital |
| 45 | L superiortemporal | R lateralorbitofrontal |
| 46 | L supramarginal | R lingual |
| 47 | L frontalpole | R medialorbitofrontal |
| 48 | L temporalpole | R middletemporal |
| 49 | L transversetemporal | R parahippocampal |
| 50 | L insula | R paracentral |
| 51 | R bankssts | R parsopercularis |
| 52 | R caudalanteriorcingulate | R parsorbitalis |
| 53 | R caudalmiddlefrontal | R parstriangularis |
| 54 | R cuneus | R pericalcarine |
| 55 | R entorhinal | R postcentral |
| 56 | R fusiform | R posteriorcingulate |
| 57 | R inferiorparietal | R precentral |
| 58 | R inferiortemporal | R precuneus |
| 59 | R isthmuscingulate | R rostralanteriorcingulate |
| 60 | R lateraloccipital | R rostralmiddlefrontal |
| 61 | R lateralorbitofrontal | R superiorfrontal |
| 62 | R lingual | R superiorparietal |
| 63 | R medialorbitofrontal | R superiortemporal |
| 64 | R middletemporal | R supramarginal |
| 65 | R parahippocampal | R frontalpole |
| 66 | R paracentral | R temporalpole |
| 67 | R parsopercularis | R transversetemporal |
| 68 | R parsorbitalis | R insula |
| 69 | R parstriangularis |  |
| 70 | R pericalcarine |  |
| 71 | R postcentral |  |
| 72 | R posteriorcingulate |  |
| 73 | R precentral |  |
| 74 | R precuneus |  |
| 75 | R rostralanteriorcingulate |  |
| 76 | R rostralmiddlefrontal |  |
| 77 | R superiorfrontal |  |
| 78 | R superiorparietal |  |
| 79 | R superiortemporal |  |
| 80 | R supramarginal |  |
| 81 | R frontalpole |  |
| 82 | R temporalpole |  |
| 83 | R transversetemporal |  |
| 84 | R insula |  |
